# Emergence of G8P[6] rotavirus strains in Korean neonates

**DOI:** 10.1186/s13099-018-0255-8

**Published:** 2018-07-03

**Authors:** Su-Kyung Lee, Seoheui Choi, Seon-Hee Shin, Eun Jin Lee, Jungwon Hyun, Jae-Seok Kim, Hyun Soo Kim

**Affiliations:** 1 Department of Laboratory Medicine, Hallym University Dongtan Sacred Heart Hospital, Hallym University College of Medicine, 7, Keunjaebong-gil, Hwaseong-si, Gyeonggi-do 18450 South Korea; 2 Department of Pediatrics, Hallym University Dongtan Sacred Heart Hospital, Hallym University College of Medicine, 7, Keunjaebong-gil, Hwaseong-si, Gyeonggi-do 18450 South Korea; 3 Department of Laboratory Medicine, Hallym University Kangdong Sacred Heart Hospital, Hallym University College of Medicine, 150, Seongan-ro, Gangdong-gu, Seoul, 05355 South Korea

**Keywords:** Rotavirus, Genotype, G8P[6], Korean, Neonate

## Abstract

**Background:**

Rotaviruses are the major causes of pediatric gastroenteritis worldwide. The genotypic distribution of rotavirus strains shows temporal and geographical fluctuations, and knowledge of the molecular epidemiology of rotaviruses is important for the development of vaccines and diagnostic reagents. We investigated VP4 and VP7 capsid genotypes of rotaviruses isolated from 211 stool specimens collected from Korean neonates in a neonatal intensive care unit from September 2017 to March 2018.

**Results:**

Of 211 stool specimens, 15 specimens (7.1%) were rotavirus-positive. Eleven specimens (73.3%) were G8P[6] type and 4 (26.7%) were G4P[6] type. Sequence analysis revealed that all G8 sequences in this study showed the highest nucleotide identity to G8 sequences of G8P[8] rotavirus strains isolated in Vietnam in 2014, and P[6] gene sequences showed the highest nucleotide identity to P[6] sequences of G4P[6] strains detected in Korea in 2012. Only one amino acid difference in VP7 was found in 3 of the 11 G8P[6] strains in this study, but multiple amino acid substitutions in VP7 were detected between these G8P[6] strains and the commonly used vaccine strains.

**Conclusions:**

This study showed that rotavirus G8P[6] strains were firstly detected at high frequency in Korean neonates from September 2017 to March 2018. These new rotavirus G8P[6] strains were estimated to be derived from reassortment events between the G8 of G8P[8] strains in Asian region and the P[6] of G4[6] in Korea. Whether the emergence of this unusual G8P[6] strain reflects continuous prevalence or transient occurrence will require continuous monitoring of rotavirus epidemiology.

## Background

Rotaviruses are the most common causes of pediatric gastroenteritis worldwide, especially in children younger than 5 years. The virus contains a genome of 11 double-stranded RNA segments surrounded by a triple-layered capsid consisting of a core, inner capsid, and outer capsid layer [1, 2]. The outer capsid layer is composed of two structural proteins, VP7 (segment 9) and VP4 (segment 4), which are targets of neutralizing antibodies. Based on VP7 and VP4 gene sequences, human group A rotaviruses are classified into G and P genotypes, and an epidemiological study has shown that at least 27 G and 37 P genotypes exist [1]. G1–G4 and G9, and P[4], P[6], and P[8] are the most frequent G and P genotypes, respectively [1, 3]. The genotypic distribution of rotavirus strains shows temporal and geographical fluctuations [4]; therefore, continuous monitoring of rotavirus molecular epidemiology is important for the development of vaccines, monitoring vaccine effectiveness, and epidemiological study of endemic and epidemic rotavirus infections.

In Korea, G1P[8] is the most frequent genotype in children, and G4P[6] is the most frequent genotype in neonates [3, 5–8]. The predominance of the G4P[6] genotype in Korean neonates has been frequently reported in several studies from 1999 to 2016 [5–9]. Interestingly, G4P[6] predominance was not reported in other countries. However, the genotype G8P[6], which has not been reported in Korean neonates, has been recently frequently detected in a neonatal intensive care unit of one hospital. In the current study, we characterized the VP7 and VP4 capsid genotypes of these new G8P[6] strains.

## Results

### Genotyping of rotavirus

The positive rate of rotavirus antigen testing in the NICU in the study period was 7.1% (15/211). Of the 15 rotavirus-positive samples, 11 (73.3%) were G8P[6] type and 4 (26.7%) were G4P[6] type (Table 1). The day of admission to the NICU when rotavirus was detected was the 1st day for 11 patients, 4th for 1 patient, 7th for 2 patients, and 30th for 1 patient (Table 1). BLAST searches of 11 G8P[6] strains revealed that all G8 sequences in this study showed the highest nucleotide identity to G8 sequences of G8P[8] rotavirus strains isolated in Vietnam in 2014 [10] (GenBank accession number: LC074743.1_G8P[8]) and P[6] gene sequences in this study showed the highest nucleotide identity to P[6] sequences of G4P[6] strains detected in Korea in 2012 (KF650090.1_G4P[6], KF650087.1_G4P[6]).

**Table 1:** Genotypes of rotaviruses detected in a NICU from September 2017 to March 2018

Patient no. (specimen no.)	Age/sex	Genotype of rotavirus	Admission day of NICU when detecting rotavirus	Route of NICU admission	Patient’s home town	Origin of rotavirus infection
1	16 days/M	G8P[6]^#^	Day 1	Transfer from A clinic	Osan	Outside
2	12 days/M	G8P[6]^#^	Day 1	Transfer from B clinic	Suwon	Outside
3	30 days/F	G8P[6]^#^	Day 30	Birth in this hospital	Pyeongtaek	NICU
4	17 days/M	G8P[6]^#^	Day 7	Transfer from C clinic	Hwaseong	NICU or outside
5	14 days/M	G8P[6]^#^	Day 1	Transfer from D clinic	Hwaseong	Outside
6	21 days/M	G8P[6]^#^	Day 1	Transfer from E clinic	Hwaseong	Outside
7	12 days/M	G8P[6]^#^	Day 1	Transfer from F clinic	Suwon	Outside
8	6 days/M	G8P[6]^#^	Day 1	Transfer from G hospital	Hwasong	Outside
9	8 days/M	G8P[6]^#^	Day 1	Transfer from H hospital	Hwasong	Outside
10	8 days/M	G8P[6]^#^	Day 1	Transfer from I clinic	Pyeongtaek	Outside
11	12 days/F	G8P[6]^#^	Day 1	Transfer from I clinic	Osan	Outside
12	14 days/M	G4P[6]	Day 1	Transfer from G hospital	Hwaseong	Outside
13	19 days/F	G4P[6]	Day 1	Transfer from G hospital	Hwaseong	Outside
14	15 days/F	G4P[6]	Day 7	Outpatient clinic in this hospital	Hwaseong	NICU or outside
15	5 days/M	G4P[6]	Day 4	Outpatient clinic in this hospital	Sungnam	NICU or outside

Positive rate of rotavirus antigen test in this period: 7.1% (15 out of 211 patients)

*NICU* neonatal intensive care unit

^#^G8P[6] strains detected in this study

### Phylogenetic and similarity analyses of rotavirus G8P[6] strains

**Fig. 1:**
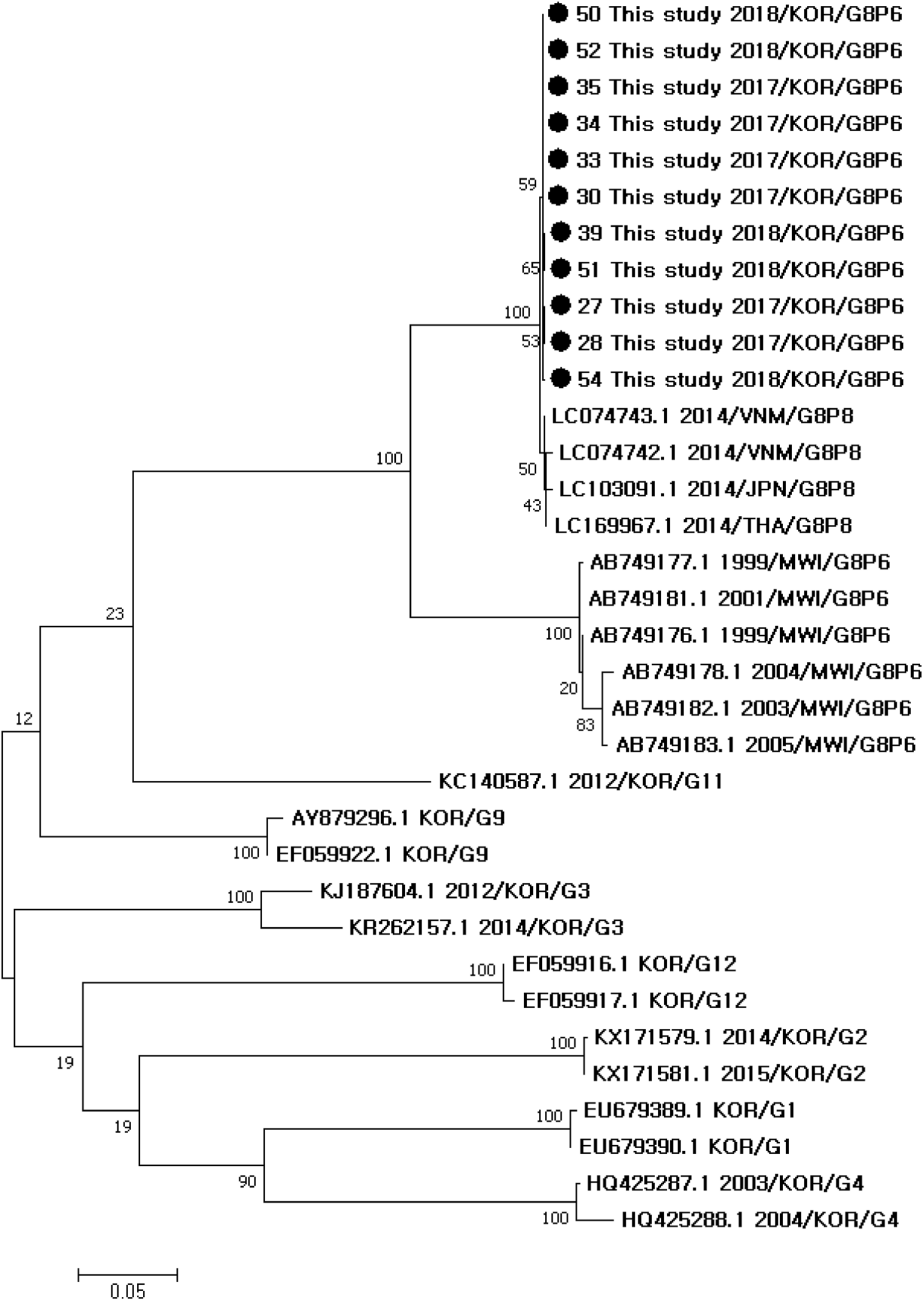
Phylogenetic tree of VP7 sequences of rotavirus G8 strains in this study and other rotavirus VP7 (G) sequences

**Fig. 2:**
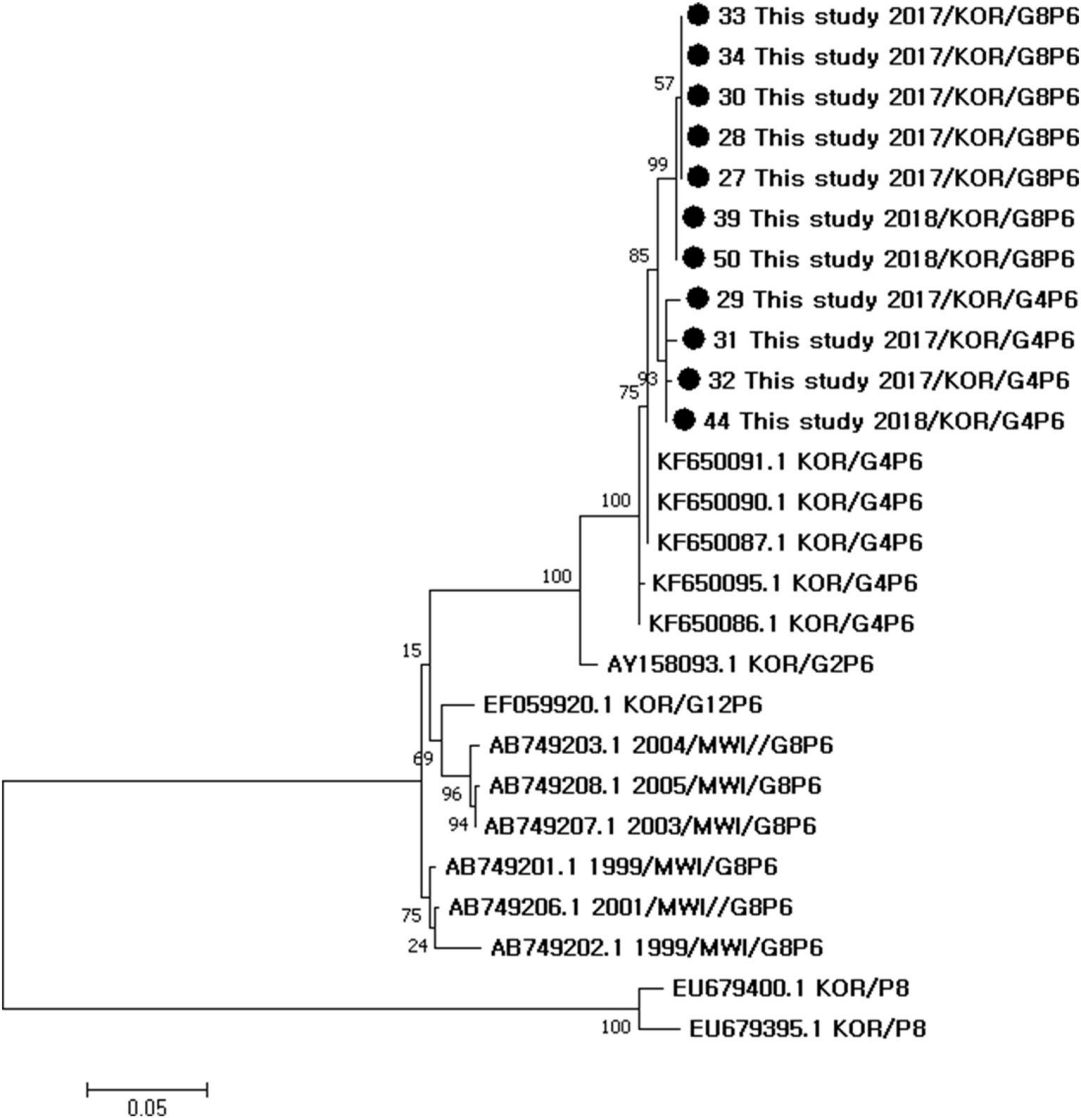
Phylogenetic tree of VP4 sequences of rotavirus P[6] strains in this study and other rotavirus VP4 (P) sequences

**Fig. 3:**
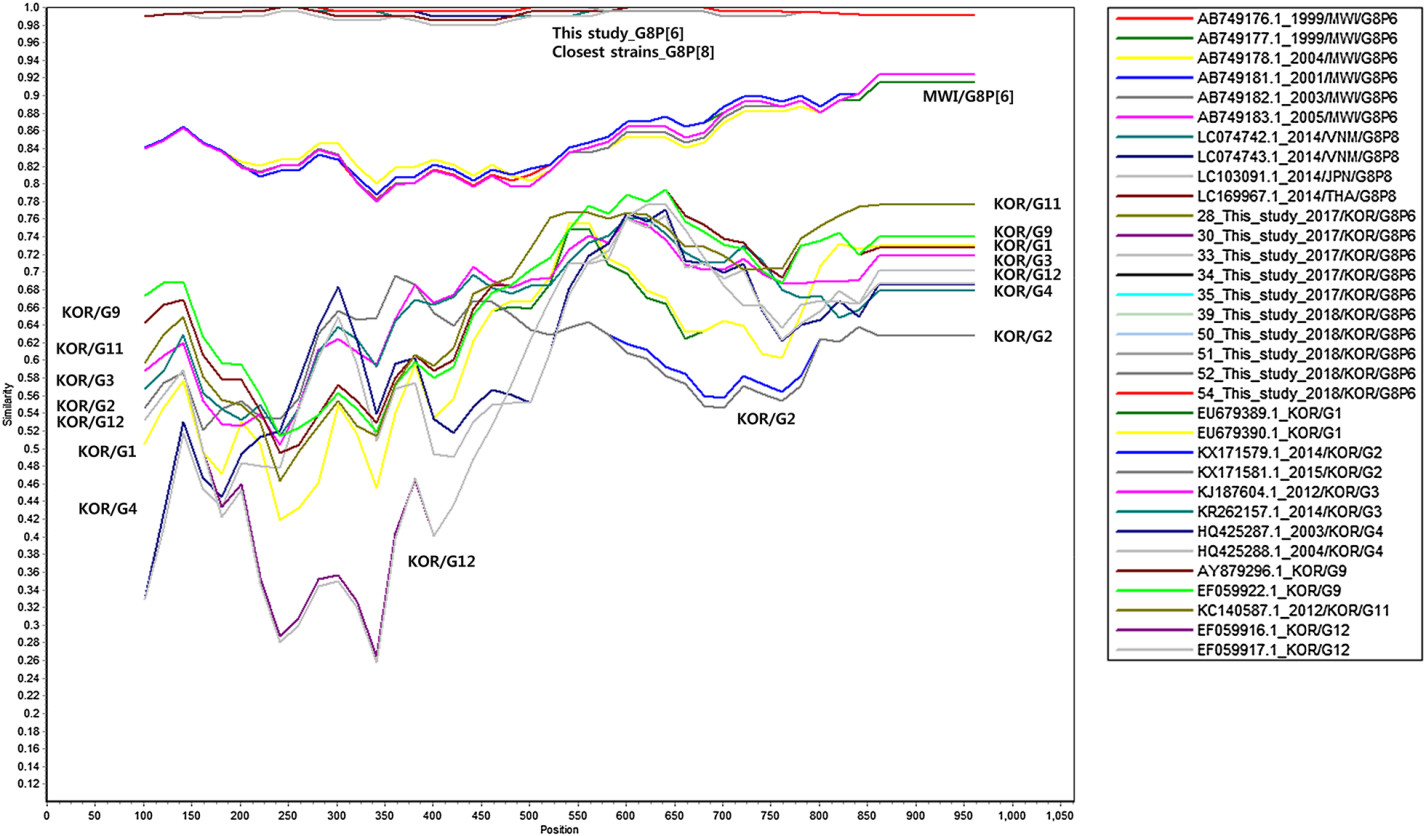
Similarity analysis of VP7 sequences of rotavirus G8 strains in this study and other rotavirus VP7 (G) sequences

Phylogenetic trees and Simplot analysis showed genetic distances among the rotavirus reference strains (Figs. 1, 2, 3). Phylogenetic analysis showed that the G8 sequences in this study were the closest to G8 of G8P[8] strains from Vietnam in 2014 (LC074743.1_G8P[8]), followed by G8 of G8P[8] from Thailand in 2014 (LC169967.1_G8P[8]) and Japan in 2014 (LC103091.1_G8P[8]), G8 of G8P[6] from Malawi [11] (AB749176.1_G8P[6], AB749177.1_G8P[6], AB749178.1_G8P[6], AB749181.1_G8P[6], AB749182.1_G8P[6], AB749183.1_G8P[6]), and then by G11, G9, G3, G12, G2, G1, and G4 strains from Korea (Fig. 1). Phylogenetic analysis showed that the P[6] sequences in this study were the closest to P[6] of G4P[6] strains from Korea (KF650091.1, KF650090.1, KF650087.1, KF650095, KF650086_G4P[6]), followed by P[6] of G2P[6] from Korea (AY150893.1_G2P[6]), P[6] of G12P[6] from Korea (EF059920.1_G12P[6]), and P[6] of G8P[6] from Malawi [11] (AB749203.1_G8P[6], AB749208.1_G8P[6], AB749207.1_G8P[6], AB749201.1_G8P[6], AB749206.1_G8P[6], AB749202.1_G8P[6]), and then by P[8] strains from Korea (EU679400.1_G1P[8] and EU679395.1_G1P[8]) (Fig. 2). Simplot analysis of VP7 genes showed that the region of first 650 nucleotides was more variable than the latter part (Fig. 3).

### Comparison of the deduced amino acids of VP7 protein of the G8P[6] strains from this study with those of reference strains

**Fig. 4:**
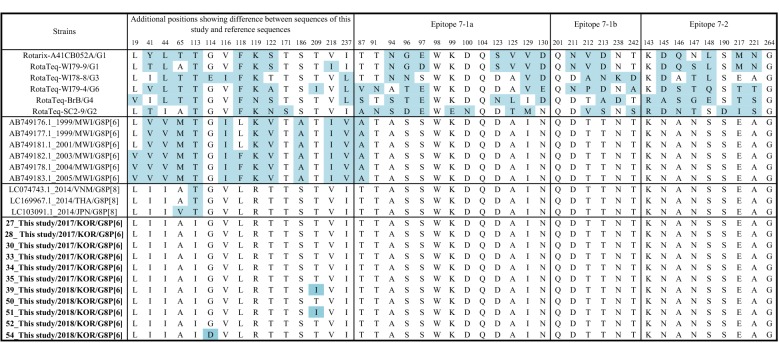
Deduced amino acid sequences of the antigenic epitopes of the VP7 (G) glycoprotein

Of the 11 G8P[6] strains included in this study, only one amino acid difference in VP7 was found in three strains (one G114D or two T209Is) (Fig. 4). Additional one amino acid difference (T113I) was detected when compared with the closest rotavirus strains from GenBank (LC074743.1_G8P[8]). No amino acid differences in epitope 7-1a, 7-1b, and 7-2 regions between the G8 sequences in this study and the closest strains (LC074743.1_G8P[8]) were detected. Multiple amino acid substitutions in VP7 were detected between G8 strains from this study and the commonly used vaccine strains (Fig. 4).

## Discussion

In this study, the rotavirus G8P[6] genotype was detected at unusually high frequency in neonates admitted to the NICU of a university hospital in Korea. This genotype had not been reported previously in Korea. Since several studies have reported that most neonatal rotavirus infections in Korea are caused by G4P[6] strains [5–9], G8P[6] emergence in neonates is remarkable and worth reporting. The G8 genotype was first detected in humans in Indonesia in 1980, and was sporadically detected worldwide, especially in Africa [12–15]. It is often detected in combination with P[6] in many countries [12, 16]. To our knowledge, this is the first report of G8P[6] rotavirus infection in Korean neonates.

The sequences of rotaviruses have changed over time. Sequence variations involve nucleotide substitution, recombination, or reassortment [17] of the 11 RNA segments. In this study, the G8 sequences of the G8P[6] strains were the closest to those of G8P[8] found in Vietnam, and the P[6] sequences of these strains were the closest to those of G4P[6] found in Korea, which suggests reassortment events occurred between the G8 of G8P[8] strain in Asia and the P[6] of G4[6] in Korea. These reassorted or variant strains may be more pathogenic to Korean neonates. Only VP4 and VP7 genes were analyzed in this study. Complete analysis of other gene segments of the rotaviruses will improve our understanding of the overall pattern of nucleotide substitution, recombination, or reassortment [17]. Additionally, we performed genotyping on only NSP5 and VP6 genes of these G8P[6] strains to investigate whether the backbone of these strains is likely to have Wa-like or DS-1-like backbone [10], and the results suggest that these G8P[6] strains appeared to have the possibility of DS-1-like genotype constellation (unpublished data).

Genetic distances between the G8 strains of this study and reference strains in GenBank were estimated through phylogenetic and Simplot analyses. Multiple amino acid substitutions in VP7 were detected between G8 strains from this study and the commonly used vaccine strains. These amino acid substitutions in rotavirus strains may be the cause of escape from host immunity obtained from Rotarix or RotaTeq vaccination.

In the present study, 9 out of 11 patients who had been infected with rotavirus G8P[6] were diagnosed with rotavirus infection on the 1st day of NICU admission, and these patients were considered to have been infected outside the NICU because rotavirus infection requires an incubation period. On the other hand, the remaining 2 patients were found to be infected on the 7th and 30th day of NICU admission, and therefore, these infections can be considered to be nosocomial within the NICU. The positive rate of rotavirus antigen testing at the NICU during the study period was 7.1%, which is lower than rotavirus infection rates reported in other studies in Korea [5, 18].

In this study, the G8 sequences of all G8P[6] showed little sequence difference, and all patients with the G8P[6] strain were born in our hospital, or transferred from nearby hospitals or postpartum care centers. Therefore, these 11 G8P[6] strains seemed to have originated from a common ancestor and spread to nearby community areas. Another possibility is that these strains are prevalent in other areas of Korea and were introduced to our hospital around the similar time. Further observation is needed as to whether this new genotype will appear and disappear in the community in short periods or will continue to propagate and disseminate.

## Conclusions

In summary, G8P[6] was frequently isolated from neonates in the NICU of one hospital in a metropolitan area of South Korea from September 2017 to March 2018. These new G8P[6] strains were estimated to be derived from a common ancestor through phylogenetic, similarity, and epitope analyses and these strains were estimated to be derived from reassortment events between the G8 of G8P[8] strains in the Asian region and P[6] of G4[6] in Korea. Whether the emergence of this unusual strain reflects continuous prevalence or transient occurrence will require continuous monitoring of rotavirus epidemiology.

## Methods

### Patient samples

Rotavirus-positive stool samples were collected from Korean neonates in a neonatal intensive care unit. Rotavirus testing was performed on the 1st day of NICU admission regardless of the presence of symptoms and when rotavirus infection was suspected. We investigated VP4 and VP7 capsid genotypes of the rotaviruses from the stool specimens. Between September 2017 and March 2018, 211 fecal specimens were tested for rotavirus antigen (SD BIOLINE Rotavirus assay, Standard Diagnostics, Korea). Of these, 15 rotavirus-positive stool samples (positive rate 7.1%) were successively collected in the neonatal intensive care unit (NICU) of Hallym University Dongtan Sacred Heart Hospital. The clinical data collected from the patients’ medical records included their age, gender, day of admission at the NICU (i.e., of rotavirus detection). Eleven (73.3%) were collected from males, and the overall median age of the donors was 14 days (range 5–30 days). This study was approved by the Institutional Review Board of Hallym University Dongtan Sacred Heart Hospital (IRB No. 2017-08-007-001).

### Rotavirus G and P genotyping

Rotavirus G (VP7) and P (VP4) genotyping was carried out using RT-PCR and sequencing. Viral RNA was extracted from fecal suspensions by using a QIAamp Viral RNA Mini kit (Qiagen, Hilden, Germany) and the QIAcube platform (Qiagen). The RNA was denatured and reverse-transcribed using the SuperScript^®^ III First-Strand Synthesis System (Invitrogen, USA). The VP7 and VP4 genes were amplified from the dsRNA genome using specific primer sets, VP7-F/VP7-R and VP4-F/VP4-R, respectively [1]. Genotyping PCR was conducted using 2.5 U AmpliTaq Gold DNA polymerase Taq (Applied Biosystems, USA). Thermal cycles included initial denaturation at 95 °C for 15 min followed by 35 cycles of 95 °C for 1 min, 52 °C (for VP7) or 50 °C (for VP4) for 1 min, and 72 °C for 1 min, and final extension at 72 °C for 10 min. The PCR products were visualized by electrophoresis in 1% agarose gel and were analyzed by DNA sequencing using ABI BigDye Terminator v3.1 Cycle Sequencing Kits (Applied Biosystems, USA) and an ABI 3500 XL DNA Analyzer (Applied Biosystems, USA). Genotypes were confirmed using the Basic Local Alignment Search Tool (BLAST) on the National Center for Biotechnology Information (NCBI) website.

### Phylogenetic and similarity analyses of rotavirus G8 strains

Phylogenetic and similarity analyses were performed to determine the relationship between most frequently detected rotavirus G8P[6] strains in this study and comparative representative VP7 (G) strains. Reference G sequences were selected from G8 sequences of complete genome assemblies of G8P[6] strains (ASM266995v1_AB749176.1_G8P[6], ASM266997v1_AB749177.1_G8P[6], ASM267003v1_AB749178.1_G8P[6], ASM267015v1_AB749181.1_G8P[6], ASM267019v1_AB749182.1_G8P[6], ASM267029v1_AB749183.1_G8P[6]), representative Korean strains (EU679389.1_G1, EU679390.1_G1, KX171579.1_G2, KX171581.1_G2, KJ187604.1_G3, KR262157.1_G3, HQ425287.1_G4, HQ425288.1_G4, EF059922.1_G9, AY879296.1_G9, KC140587.1_G11, EF059916.1_G12, EF059917.1_G12), and the closest ones with G8 sequences obtained in this study (LC074743.1_G8P[8], LC103091.1_G8P[8], LC169967.1_G8P[8]). Reference P[6] sequences were selected from P[6] sequences of complete genome assemblies of G8P[6] strains from Malawi [11] (AB749203.1_G8P[6], AB749208.1_G8P[6], AB749207.1_G8P[6], AB749201.1_G8P[6], AB749206.1_G8P[6], AB749202.1_G8P[6]), representative Korean P[6] strains (KF650091.1, KF650090.1, KF650087.1, KF650095, KF650086_G4P[6]; AY150893.1_G2P[6]; EF059920.1_G12P[6]), and P[8] strains from Korea (EU679400.1_G1P[8] and EU679395.1_G1P[8]). Representative G8 and P[6] sequences were aligned with MEGA version 7 [19]. Phylogenetic trees were constructed using the maximum likelihood method and Tamura–Nei substitution models with 1000 bootstrap replications. The percentages of similarity between sequences and reference sequences were assessed with the SimPlot program version 3.5.1.

### Comparison of the VP7 capsid protein antigenic epitopes of the strains from this study with those of reference strains

Deduced amino acid sequences of rotavirus VP7 capsid antigens were analyzed and were compared to the representative reference G8 sequences. The rotavirus VP7 protein contains two antigenic epitopes (7-1 and 7-2), which are responsible for evoking neutralizing antibodies, and epitope 7-1 is further subdivided into 7-1a and 7-1b [5, 20]. The capsid hypervariable antigenic epitopes (7-1a, 7-1b, 7-2) of the rotavirus strains obtained in this study were compared to those of reference strains and rotavirus vaccine strains (Rotarix-A41CB052A_G1, RotaTeq-WI79-9_G1, RotaTeq-WI78-8_G3, RotaTeq-WI79-4_G6, RotaTeq-BrB_G4, RotaTeq-SC2-9_G2). Multiple sequence alignment and translation to amino acids were conducted using the MEGA version 7 program.

## Abbreviations

NCBI: National Center for Biotechnology Information
BLAST: Basic Local Alignment Search Tool
NICU: neonatal intensive care unit
RT-PCR: reverse transcription-polymerase chain reaction
